# Emergence of norovirus GII.P16-GII.2 strains in patients with acute gastroenteritis in Huzhou, China, 2016–2017

**DOI:** 10.1186/s12879-018-3259-6

**Published:** 2018-07-24

**Authors:** Jiankang Han, Xiaofang Wu, Liping Chen, Yun Fu, Deshun Xu, Peng Zhang, Lei Ji

**Affiliations:** Huzhou Center for Disease Control and Prevention, 999 Changxing Road, Huzhou, 313000 Zhejiang China

**Keywords:** Norovirus, GII.P16-GII.2, Molecular epidemiology, Acute gastroenteritis

## Abstract

**Background:**

In late 2016, an uncommon recombinant NoV genotype called GII.P16-GII.2 caused a sharp increase in outbreaks of acute gastroenteritis in different countries of Asia and Europe, including China. However, we did not observe a drastic increase in sporadic norovirus cases in the winter of 2016 in Huzhou. Therefore, we investigate the prevalence and genetic diversity of NoVs in the sporadic acute gastroenteritis (AGE) cases from January 2016 to December 2017 in Huzhou City, Zhejiang, China.

**Methods:**

From January 2016 to December 2017, a total of 1001 specimens collected from patients with AGE were screened for NoV by real-time RT-PCR. Partial sequences of the RNA-dependent RNA polymerase (RdRp) and capsid gene of the positive samples were amplified by RT-PCR and sequenced. Genotypes of NoV were confirmed by online NoV typing tool and phylogenetic analysis. Complete VP1 sequences of GII.P16-GII.2 strains detected in this study were further obtained and subjected into sequence analysis.

**Results:**

In total, 204 (20.4%) specimens were identified as NoV-positive. GII genogroup accounted for most of the NoV-infected cases (98.0%, 200/204). NoV infection was found in all age groups tested (< 5, 5–15, 16–20, 21–30, 31–40, 41–50, 51–60, and >60 years), with the 5–15 year age group having the highest detection rate (17/49, 34.7%). Higher activity of NoV infection could be seen in winter-spring season. The predominant NoV genotypes have changed from GII.Pe-GII.4 Sydney2012 and GII.P17-GII.17 in 2016 to GII.P16-GII.2, GII.Pe-GII.4 Sydney2012 and GII.P17-GII.17 in 2017. Phylogenetic analyses revealed that 2016–2017 GII.P16-GII.2 strains were most closely related to Japan 2010–2012 cluster in VP1 region and no common mutations were found in the amino acids of the HBGA-binding sites and the predicted epitopes.

**Conclusions:**

We report the emergence of GII.P16-GII.2 strains and characterize the molecular epidemiological patterns NoV infection between January 2016 and December 2017 in Huzhou. The predominant genotypes of NoV during our study period are diverse. VP1 amino acid sequences of 2016–2017 GII.P16-GII.2 strains remain static after one year of circulation.

**Electronic supplementary material:**

The online version of this article (10.1186/s12879-018-3259-6) contains supplementary material, which is available to authorized users.

## Background

Noroviruses (NoVs) are the second most common cause of viral acute gastroenteritis cases worldwide, affecting people in all age groups. It is estimated that NoVs account for 18% of sporadic cases of acute gastroenteritis and approximately 50% of all-cause gastroenteritis outbreaks worldwide [1, 2].

NoV is a member of the family Caliciviridae. The viral genome is a single positive-strand RNA of 7.7 kb that contains three open reading frames (ORFs) [3]. ORF1 encodes a nonstructural polyprotein required for replication, including RNA-dependent RNA polymerase (RdRp). ORF2 encodes the major capsid protein (VP1) and ORF3 encodes a minor capsid protein (VP2). VP1 is structurally divided into a shell (S) and a protruding (P) domain [4]. The P domain is further divided into P1 and the hypervariable P2 subdomain with the major antigenic sites and histoblood group antigen (HBGA) ligands located in the P2 subdomain [5]. NoV are a highly diverse group of viruses that can be genetically grouped into 7 genogroups (GI–GVII), but only norovirus GI, GII, and GIV can infect humans, with GII being the most prevalent [6, 7]. Each genogroup can be further classified into numerous genotypes based on the sequence differences of their VP1 proteins. To date, 9, 22 and 2 capsid genotypes have been recognized in GI, GII and GIV.

Despite this diversity, the NoV GII.4 virus has been responsible for most reported norovirus-associated outbreaks and sporadic cases for over two decades globally. New variants of GII.4 strains emerge every 2–3 years [8, 9]. Since 2008, almost all outbreaks and the majority of sporadic cases in Huzhou were caused by GII.4 variants, including GII.4 2006b, New Orleans 2009, and Sydney2012 [10, 11]. However, during the winter of 2014–2015, a novel GII.17 NoV strain emerged and replaced the previously dominant GII.4 genotype Sydney2012 variant, as the major cause of acute gastroenteritis outbreaks in several countries of the Asia [12]. At the same time, GII.17 emerged and became the dominant strains during the 2014–2015 epidemic season in Huzhou [13]. This was the first time that non–GII.4 norovirus might become the predominant genotype. Moreover, in late 2016, an uncommon recombinant NoV genotype called GII.P16-GII.2 caused a sharp increase in outbreaks of acute gastroenteritis in many cities of China, most of the outbreaks (78%) associated with this recombinant variant occurred in kindergartens [14]. Increased circulation of GII.P16-GII.2 recombinant was also observed concurrently in Japan, Germany and France during the same winter season [15–17]. However, we did not observe a drastic increase in sporadic NoV cases in the winter of 2016 in Huzhou. Whether GII.P16-GII.2 recombinant has emerged and been prevalent in Huzhou is unclear. Therefore, we investigate the prevalence and genetic characteristics of NoVs in the sporadic acute gastroenteritis (AGE) cases from January 2016 to December 2017 in Huzhou City, Zhejiang, China.

## Methods

### Specimen collection

An ongoing hospital-based local NoV gastroenteritis surveillance program has been conducted at the First People’s Hospital in Huzhou since January 2013. Surveillance subjects were those who visited the enteric disease clinics and presented with clinical symptoms of AGE. The definition of AGE was diarrhea (three or more loose or watery stools within 24 h), possibly accompanied by vomiting, abdominal pain, fever, and nausea. This study was part of the regional NoV gastroenteritis surveillance program and was approved by the human research ethics committee of Huzhou Center for Disease Control and Prevention. Stool samples were freshly collected from surveillance subjects between January 2016 and December 2017 for routine NoV detection. Informed consent for the stool samples was obtained from the patients or their guardians.

### Viral RNA extraction and Nov detection

Viral RNA was extracted from 10% stool-PBS suspensions with a QIAamp viral RNA mini kit (Qiagen, Hilden, Germany) according to the manufacturer’s instructions. NoV detection was performed by real-time reverse transcription polymerase chain reaction (RT-PCR) using genogroup-specific primers and probes (JJV1F/JJV1R/JJV1P and JJV2F/COG2R/RING2-TP) as previously described [10, 18] . Real-time RT-PCR was carried out using a One Step PrimeScript® RT-PCR Kit (DRR064, TaKaRa, Dalian, China).

### Genomic amplification for genotyping

NoV positive specimens were genotyped based on partial sequence of RdRp (region A in ORF1) and VP1 genes (region C in ORF2) as previously described [10]. Primer set JV12Y/JV13I was used for RdRp typing [19]. Primer set G1SKF/G1SKR and G2SKF/G2SKR were used for VP1 typing for GI and GII, respectively [20]. Fragment spanning the ORF1/ORF2 overlap region was amplified using the primers JV12Y and G1SKR/G2SKR. To identify potential antigenic mutations, primers VP1F/VP1R (Additional file 1: Table S1) were designed and used for full-length VP1 amplification of NoV GII.P16-GII.2 strains. RT-PCR was carried out using TaKaRa One-Step RT-PCR Kit (DRR024, TaKaRa, Dalian, China). RT-PCR conditions were as follows: 42 °C for 30 min followed by 95 °C for 2 min, and 35 cycles of PCR at 94 °C for 30 s, at 48 °C for 30 s, at 72 °C for 90 s, and a final incubation at 72 °C for 10 min. After amplification, the PCR products were visualized by electrophoresis and direct sequenced by TaKaRa Biotechnology (Dalian, China).

### Phylogenetic analysis and molecular genotyping

All nucleotide sequences were initially genotyped using the RIVM online norovirus genotyping tool (http://www.rivm.nl/mpf/norovirus/typingtool). The phylogenetic tree was constructed using the neighbor-joining method with MEGA software (version 6.06) [21] and bootstrap analysis was performed with 1000 replications.

### Nucleotide sequence accession numbers

The sequences of the NoV strains obtained in this study were deposited in the GenBank under the accession numbers KY344281to KY344283, KY344303 to KY344328, MG739472 to MG739503, KY344334 to KY344337, KY344368 to KY344403, MG763290 to MG763293, MG763311 to MG763353, MG763402 to MG763454, and MG763365 to MG763377.

## Results

### Prevalence of NoV infections

**Fig. 1:**
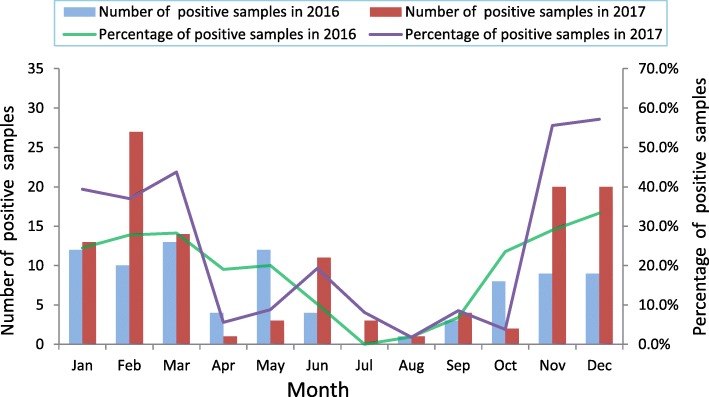
Monthly distribution of NoV infections from January 2016 to December 2017. The bar and lines charts represents the number and the percentage of NoV positive samples in each month during the study period

From January 2016 to December 2017, a total of 1001 specimens (491 in 2016, 510 in 2017) collected from patients with AGE were screened for NoV by real-time RT-PCR. In total, 204 (20.4%) specimens were identified as NoV-positive. The positive rate of NoV infection was 17.3% (85/491) and 23.3% (119/510) in 2016 and 2017 respectively. GII genogroup accounted for most of the NoV-infected cases (98.0%, 200/204). Infection with NoV was found in all age groups tested (Table 1). The highest detection rate was in the 5–15 year age group (17/49, 34.7%), followed by 41–50 years (24/100, 24.0%), 31–40 years (41/ 176, 23.3%), 21–30 years (56/256, 21.9%), 51–60 years (26/122, 21.3%), 16–20 years (19/96, 19.8%), <5 years (6/45, 13.3%) and >60 years (15/157, 9.6%). The monthly distribution of NoV infections during our study period is shown in Fig. 1. Higher activity of NoV infection could be seen in winter-spring season (from November to March), the detection rate was all >20.0%. In contrast, lower activity of NoV infection were observed in summer-autumn (from July to September), when the average detection rate was only 4.5%.

**Table 1:** The detection of NoVs in AGE patients of different ages

Parameter	Tested cases (*N* = 1001)	Positve cases (*N* = 204)	Negative cases (*N* = 797)	Positive rate	P
Age					0.004
< 5	45	6	39	13.3%	
5–15	49	17	32	34.7%	
16–20	96	19	77	19.8%	
21–30	256	56	200	21.9%	
31–40	176	41	135	23.3%	
41–50	100	24	76	24.0%	
51–60	122	26	96	21.3%	
>60	157	15	142	9.6%

### Distribution and clinical features of NoV genotypes

Of the 204 NoV-positive samples confirmed by real-time RT-PCR, 140 samples were successfully sequenced and genotyped by RT-PCR (Table 2). Partial nucleotide sequences of both RdRp and capsid were obtained for 120 strains, which clustered into 17 genotypes, including GI.Pb-GI.6, GI.P7-GI.7, GII.Pe-GII.4 Sydney2012, GII.P17-GII.17, GII.P12-GII.3, GII.P16-GII.13, GII.P7-GII.6, GII.Pe-GII.4 untypable, GII.P21-GII.13, GII.P17-GII.13, GII.P21-GII.17, GII.Pe-GII.17, GII.P2-GII.2, GII.P16-GII.4 Sydney2012, GII.P7-GII.14, GII.P4 2006b-GII.4 untypable. Of the total number of strains genotyped with two genes, 75.8% (91/120) corresponded to recombinant strains. The top 3 circulating genotypes during the study period were GII.Pe-GII.4 Sydney2012 (39/140, 27.9%), GII.P16-GII.2 (29/140, 20.7%), GII.P17-GII.17 (27/140, 19.3%), followed by GII.P12-GII.3, GII.P16-GII.13 and other genotypes. Furthermore, 20 strains could only be genotyped based on partial capsid gene, including GI.6, GII.2, GII.3, GII.4 Sydney2012, GII.6, GII.13, and GII.17.

**Table 2:** Genotype distribution of identified NoV strains in Huzhou, 2016–2017

Genotype	2016	2017	total
	Number(percentage)	Number(percentage)	Number(percentage)
RdRp/Capsid			
GI.Pb-GI.6	1(1.60%)	0	1(0.70%)
GI.P7-GI.7	1(1.60%)	0	1(0.70%)
GII.Pe-GII.4 Sydney2012	18(28.60%)	21(27.30%)	39(27.90%)
GII.P17-GII.17	16(25.40%)	11(14.30%)	27(19.30%)
GII.P12-GII.3	4(6.30%)	3(3.90%)	7(5.00%)
GII.P16-GII.13	4(6.30%)	0	4(2.90%)
GII.P7-GII.6	1(1.60%)	0	1(0.70%)
GII.Pe-GII.4 untypable	2(3.20%)	0	2(1.40%)
GII.P21-GII.13	1(1.60%)	0	1(0.70%)
GII.P17-GII.13	1(1.60%)	0	1(0.70%)
GII.P21-GII.17	1(1.60%)	0	1(0.70%)
GII.Pe-GII.17	1(1.60%)	0	1(0.70%)
GII.P2-GII.2	1(1.60%)	0	1(0.70%)
GII.P16-GII.2	1(1.60%)	28(36.40%)	29(20.70%)
GII.P16-GII.4 Sydney2012	0	2(2.60%)	2(1.40%)
GII.P7-GII.14	0	1(1.30%)	1(0.70%)
GII.P42006b-GII.4 untypable	0	1(1.30%)	1(0.70%)
Capsid			
GI.6	2(3.20%)	0	2(1.40%)
GII.3	3(4.80%)	5(6.50%)	8(5.70%)
GII.6	1(1.60%)	0	1(0.70%)
GII.13	1(1.60%)	0	1(0.70%)
GII.17	3(4.80%)	0	3(2.10%)
GII.2	0	2(2.60%)	2(1.40%)
GII.4 Sydney2012	0	3(3.90%)	3(2.10%)
Total	63(100.00%)	77(100.00%)	140(100.00%)

**Fig. 2:**
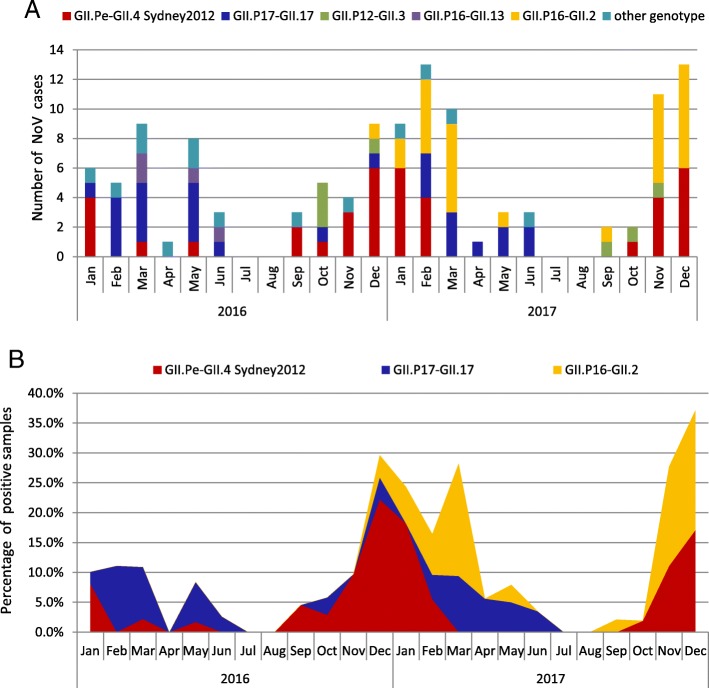
Distribution of NoV genotypes detected according to month. (**a**) The bar chart represent the number of NoV cases genotyped as GII.Pe-GII.4 Sydney2012, GII.P17-GII.17, GII.P12-GII.3, GII.P16-GII.13, GII.P16-GII.2 and other genotypes in each month during the study period. (**b**) The stacked area chart represents the percentage of NoV positive samples genotyped as GII.Pe-GII.4 Sydney2012, GII.P17-GII.17 and GII.P16-GII.2 in each month during the study period

The predominant genotypes of NoV during our study period are diverse according to month. The number of NoV cases genotyped as GII.Pe-GII.4 Sydney2012, GII.P17-GII.17, GII.P12-GII.3, GII.P16-GII.13, GII.P16-GII.2 and other genotype in each month is shown in Fig. 2a. In 2016, GII.17 was predominantly detected from February to June, accounting for 50.0% (13/26) of genotyped strains, followed by GII.P16-GII.13 (15.3%, 4/26). In contrast, GII.4 Sydney2012 was the predominant type in January and September–December, accounting for 61.5% (16/26), followed by GII.P12-GII.3 (15.4%, 4/26), GII.P17-GII.17 ranked third (11.5%, 3/26).

The percentage of NoV cases genotyped as GII.Pe-GII.4 Sydney2012, GII.P17/GII.17 and GII.P16-GII.2 in each month is shown in Fig. 2b. NoV GII.P16-GII.2 strain was first detected in December 2016 and kept increasing in early 2017. The percentage of GII.P16-GII.2 cases increased from 3.70% in November 2016 to 18.8% in March 2017. By February 2017, GII.P16-GII.2 outnumbered GII.4 Sydney2012 and it went on to replace GII.4 Sydney2012 as the predominant genotype from February to March and November to December. During the same period, we observed a higher NoV activity than those of the previous year in our surveillance (NoV detection rate 2016 vs. 2017: 24.5% vs. 39.4% in January, 27.8% vs. 36.9% in February, 28.2% vs. 43.8% in March, 29.0% vs. 55.6% in November, 33.3% vs. 57.1% in December). In contrast, the percentage of GII.4 cases decreased since January 2017; it then re-emerged in October and became second dominant genotype from November to December 2017. As for GII.P17-GII.17, it still can be detected in 2017 and seemed to be dominant between April to June when GII.P16-GII.2 and GII.4 Sydney2012 were all at low activity.

We also compared the clinical characteristics of NoV infection caused by GII.P16-GII.2, GII.P17-GII.17 and GII.Pe-GII.4 Sydney2012 in the AGE patients (Table 3). No significant differences were found in terms of clinical features, which may be due to the limited number of cases in our study. Among the 29 patients infected with GII.P16-GII.2 virus, the most common clinical manifestation was watery stool (28/29, 96.6%), followed by abdominal pain (22/29, 75.9%), vomiting (8/29, 27.6%) and fever (2/29, 6.9%).

**Table 3:** Clinical characteristics of NoV infection caused by GII.P16-GII.2, GII.P17-GII.17 and GII.Pe-GII.4 Sydney2012 in the AGE patients

Parameter	GII.P16-GII.2 (*N* = 29)	GIIP17-GII17 (*N* = 27)	GII.Pe-GII.4 Sydney2012 (*N* = 39)	P
Fever (> 38 °C)	2(6.9%)	0	2(5.1%)	0.409
Vomiting	8(27.6%)	5(18.5%)	6(15.4%)	0.449
Watery Stool	28(96.6%)	25(92.6%)	38(97.4%)	0.61
Diarrhea (times/day)				0.899
3–4	19(65.5%)	18(66.7%)	24(61.5%)	
≥5	10(34.5%)	9(33.3%)	15(38.5%)	
Abdominal pain	22(75.9%)	21(77.8%)	28(71.8%)	0.848
Inpatient	1(3.4%)	0	2(5.1%)	0.501

### Phylogenetic analyses of GII.P16-GII.2 strains

**Fig. 3:**
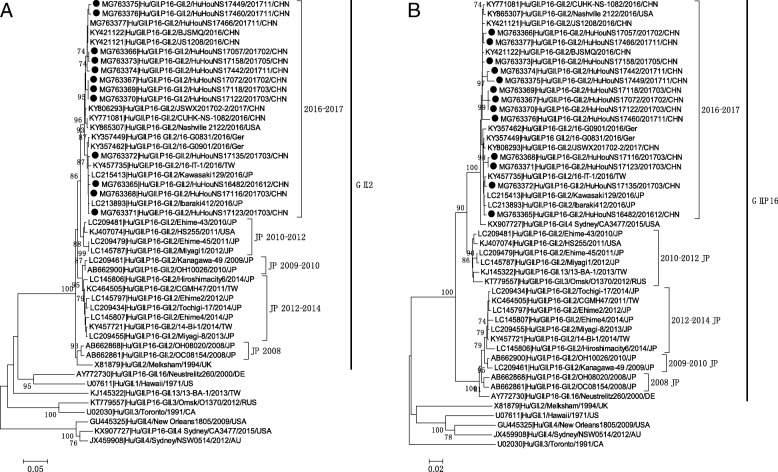
Phylogenetic analyses based on complete VP1 sequences (**a**) and partial RdRp sequences (**b**) of GII.P16-GII.2 strains. The trees were generated using the neighbor-joining method, validated by 1000 bootstrap replicates. Bootstrap values ≥ 70% are shown on the branch. GII.P16-GII.2 strains identified in this study are indicated by closed circles. All sequences names are formatted as GenBank accession number|host/genotype/sample name/isolate time/country

Using overlapping primers JV12Y/ G2SKR and VP1F/VP1R, partial RdRp and complete VP1 sequences were further obtained from 13 GII.P16-GII.2 strains (accession numbers: MG763365 to MG763377) detected from December 2016 to November 2017 in Huzhou. Analysis revealed that RdRp and VP1 sequences of the 13 GII.P16-GII.2 strains share higher degree nucleotide identity (97.3~ 100%) and amino acid identity (98.9~ 100%). Phylogenetic analysis of complete VP1 sequence showed that GII.P16-GII.2 strains in this study were clustered with GII.P16-GII.2 strains detected by the end of 2016 in China, Germany and Japan, and formed a relatively independent subcluster (designated as 2016–2017 subcluster in Fig. 3a). This subcluster was most closely related to pre-2016 GII.P16-GII.2 strains identified in Japan and US during 2010–2012 (designated as Japan 2010–2012 subcluster). The other pre-2016 GII.P16-GII.2 strains which were mainly detected in Japan after 2008 could be subdivided into three subclusters of strains in 2008, 2009–2010 and 2012–2014. Phylogenetic analysis based on partial RdRp sequences showed that 2016–2017 GII.P16-GII.2 strains also clustered together but were separated from Japan 2010–2012 strains, and genetically closely related to the GII.P16-GII.4 strain identified in 2015 (Fig. 3b).

**Fig. 4:**
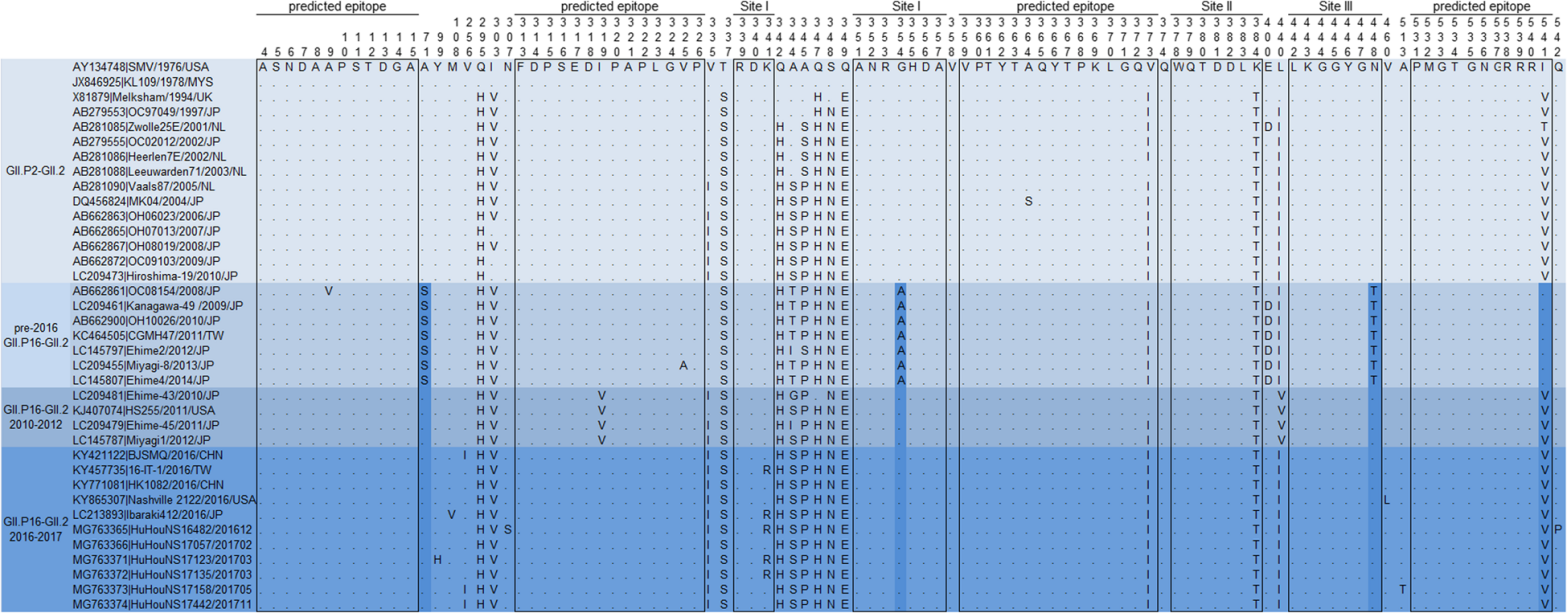
Amino acid variations in the VP1 protein of NoV GII.P16-GII.2 strains. The conserved HBGA-binding interfaces and predicted epitopes in GII.2 are indicated by empty rectangles. GII.P16-GII.2 strains identified in Huzhou are designated by accession number, sample number, isolate time. Reference sequences are indicated by their accession number, strain name, year and country

To identify potential antigenic mutations, 41 complete capsid protein VP1 sequences of NoV GII.2 strains from 1976 to 2017 were aligned, including 6 GII.P16-GII.2 sequences we determined in this study (Fig. 4). Sequence alignment analysis shows that VP1 amino acid sequences of 2016–2017 GII.P16-GII.2 strains remain almost invariable after one year of circulation in the human population. Compared with the earliest GII.2 strain (SMV/1976/USA) available in GenBank, 22 amino acid mutations were found in the VP1 region of 2016–2017 GII.P16-GII.2 strains, no mutations were found in the HBGA-binding sites [22] and the predicted epitopes [23] of NoV GII.2 except one unique amino acid change at position 341(_K_341_R_), which is part of the HBGA-binding site I. It is noteworthy that mutations at point 341 are not common to all 2016–2017 GII.P16-GII.2 strains. Furthermore, 4 Sites in VP1 shared the same residue (_A_71, _G_354, _N_448 and _V_541) between the 2016–2017 GII.P16-GII.2 strains and Japan 2010–2012 strains, which can be used to differentiate with the other pre-2016 GII.P16-GII.2 strains detected after 2008.

## Discussion

In this study, we analyzed NoV-associated sporadic acute gastroenteritis in Huzhou from January 2016 to December 2017. Our data showed that NoV infection was detected throughout the year, and the circulation peak occurred during the winter–spring seasons. NoV strains circulate in Huzhou exhibits considerable genetic diversity during the study period and at least 17 genotypes were identified based on both RdRp and capsid genes, with most of them were recombinant strains. The predominant NoV genotypes have changed from GII.Pe-GII.4 Sydney2012 and GII.P17-GII.17 in 2016 to GII.P16-GII.2, GII.Pe-GII.4 Sydney2012 and GII.P17-GII.17 in 2017. NoV GII.P16-GII.2 was first detected in December 2016 and rapidly became the major genotype in the Huzhou by February 2017, result in increasing detection rates of NoV infection in spring–winter seasons of 2017. It should be noted that this time point was later than the first report of NoV GII.P16-GII.2 in China. NoV GII.P16-GII.2 emerged first in August 2016 in China, it then caused a steep rise in gastroenteritis outbreaks at the end of 2016 in many cities of China [14, 24, 25]. However, our monitoring data showed that only one GII.P2-GII.2 caused gastroenteritis outbreak was detected in December in the winter of 2016 in Huzhou (data not shown). Similar finding were reported Jiangsu provinces, in which the NoV GII.P16-GII.2 genotype firstly appeared in December of 2016, and spread extensively in February and caused rapidly increased outbreaks in the late spring of 2017 [26]. Based on the above results, we speculate that the beginning time of GII.P16-GII.2 prevalence in different areas of China is not exactly the same.

For over two decades, most NoV-associated gastroenteritis cases reported all over the world have been linked to a single genotype GII.4 [8]. However, in winter 2014–2015, the GII.4 strains were unexpectedly displaced by a new GII.17 variant (GII.P17-GII.17 Kawasaki) in some Asian countries [12]. Moreover, in late 2016, an uncommon recombinant NoV genotype called GII.P16-GII.2 caused a sharp increase in outbreaks in different countries worldwide [14–17]. This was another event that non-GII.4 NoV might become the predominant genotype. Unlike epidemic pattern observed in the past years for GII.4 in Huzhou that new GII.4 variants emerged every two or three years and presented absolutely predominant during norovirus epidemic seasons [10, 11], these non-GII.4 predominant genotypes were co-circulating with GII.4 and the predominant genotypes of NoV are diverse according to month. In the Huzhou area, GII.17 was firstly detected in October 2014 and gradually replaced GII.4 Sydeny2012 as a predominant strain [27]. However, GII.4 Sydney2012 re-emerged in October 2015 [13] and presented an alternate prevalence pattern with GII.17 until the advent of GII.P16-GIII.2 at the end of 2016. In 2017, GII.P16-GII.2 was co-circulating with GII.Pe-GII.4 Sydney2012 and GII.P17-GII.17. A recent report showing that the non–GII.4 NoV remains static may explain the epidemiological pattern of non–GII.4 NoV infection [28]. The persistence and predominance of GII.4 strains due to it is an “evolving genotype” which can continuous evolve through accumulation of mutations into antigenically distinct variant that escape from herd immunity. In contrast, non-GII.4 predominant genotypes would prevail for only a short period of time as they are highly conserved or “static genotypes”, which limits their prevalence and antigenic diversity.

Globally, GII.P16-GII.2 is an uncommon genotype with low detection rate in sporadic infections before 2016, except in Japan. GII.P16-GII.2 was firstly reported in 2008 and became the most prevalent GII.2 genotype after 2009 in Japan [29–31]. Most of the pre-2016 GII.P16-GII.2 strains were detected in Japan during 2009–2014, which could be genetically subdivided into three clusters (2009–2010 cluster, 2010–2012 cluster, and 2012–2014 cluster) [31]. Phylogenetic analyses in this study indicated that the VP1 genes of 2016–2017 GII.P16-GII.2 strains were most closely related to Japan 2010–2012 cluster, whereas the RdRp genes of these strains were genetically closely related to the GII.P16-GII.4 strain identified in 2015. This finding is consistent with a recent phylogenetic study conducted in Japan [32]. The authors suggested that the VP1 of 2016 GII.P16-GII.2 strains diverged from common ancestors of the GII.P16-GII.2 cluster detected in 2010–2012 and RdRp of the 2016 strains diverged from the common ancestors of GIIP16-GII.4.

The driving force behind the rapidly spread of GII.P16-GII.2 stains in 2016–2017 season is not clear yet. Complete VP1 amino acid sequences of 2016–2017 GII.P16-GII.2 stains were aligned with GII.2 strains from 1976 to 2015 in this study. Compared with the earliest GII.2 strain(SMV/1976/USA), no common mutations were found in the amino acids of the HBGA-binding sites and the predicted epitopes, in concordance with the relatively static nature of non-GII.4 stains [28]. Just recently, Tohma K et al. investigated the evolutionary dynamics of GII.2 strains by using bioinformatics analysis [33]. They found the reemergence of GII.P16-GII.2 strains during 2016 did not involve changes in the substitution rate or acquisition of amino acid mutations in the major capsid protein and suggested that, genetic change in the RdRp may play some role in the predominance of GII.P16-GII.2 strains. However, the above study is only about the sequence analysis of the RdRp and VP1 region, further analysis using long sequences covered other nonstructural proteins and VP2 and in vitro assays such as serum inhibition/neutralization tests are required to explore the mechanisms behind the rapidly spread of GII.P16-GII.2 stains.

Our study is limited by a small sample size, single-site setting, and especially the partial genotyping of circulating NoVs. Genotyping was only successful for 140 (68.7%) detected NoVs cases. In future studies, epidemiologic and virologic surveillance should be broadened to better clarify the epidemiological patterns and genetic characteristics of NoV strains in Huzhou.

## Conclusion

In conclusion, we report the emergence of GII.P16-GII.2 strains and characterize the molecular epidemiological patterns NoV infection between January 2016 and December 2017 in Huzhou. We found that the predominant genotypes of norovirus during our study period in Huzhou are diverse according to month. Phylogenetic analyses showed that VP1 genes of 2016–2017 GII.P16-GII.2 strains were most closely related to Japan 2010–2012 cluster and their VP1 amino acid sequences remain relatively static after one year of circulation in the human population. However, unlike the recently emerged GII.P17-GII.17 strains that predominated only in part of Asia in 2014–2015 season, the re-emerged GII.P16-GII.2 strains were almost simultaneously reported in Asia and Europe, indicating the fast spread of this genotype across continents. Continuous surveillance and further studies are needed to understand the epidemiological characteristics of GII.P16-GII.2 strains and explore the mechanisms behind the wide spread in population of this genotype.

## Additional file

Additional file 1: **Table S1.** Primers used to amplify the complete VP1 gene of GII.P16/GII.2 NoV. (DOCX 30 kb)

## Abbreviations

AGE: Acute gastroenteritis
GI: Genogroup I
GII: Genogroup II
NoV: Norovirus
ORF: Open reading frames
RdRp: RNA-dependent RNA polymerase
RT-PCR: Reverse transcription polymerase chain reaction
