# Supplementary material for: Emergence of norovirus GII.P16-GII.2 strains in patients with acute gastroenteritis in Huzhou, China, 2016–2017
Source: BMC Infect Dis. 2018 Jul 24;18:342. doi: 10.1186/s12879-018-3259-6 (PMC6056945; doi:10.1186/s12879-018-3259-6)
Supplement: Supplementary file 1 — Table S1. Primers used to amplify the complete VP1 gene of GII.P16/GII.2 NoV. (DOCX 30 kb) [file 12879_2018_3259_MOESM1_ESM.docx]

Table S1 Primers used to amplify the complete VP1 gene of GII.P16/GII.2 NoV

| Primer name | Sequence (5′→3′) | Position |
| --- | --- | --- |
| VP1F | TCAGYACCGACATAAAAT | 4611-4628 |
| VP1R | CTGCAAGACCAGCTACAA | 6722-6742 |

# Corresponding nucleotide position of HK/2016/GII.P16_GII.2/CUHK-NS-1082 (accession no. KY771081)
